# Supplementary material for: Hybrid Models and Biological Model Reduction with PyDSTool
Source: PLoS Comput Biol. 2012 Aug 9;8(8):e1002628. doi: 10.1371/journal.pcbi.1002628 (PMC3415397; doi:10.1371/journal.pcbi.1002628)
Supplement: Text S4 — Complete source code for the PyDSTool package (version 0.88.120504). Includes API documentation and help files linking to web pages. This file is identical to the current public release on Sourceforge.net. (ZIP) [file pcbi.1002628.s004.zip › PyDSTool/html/PyDSTool.Toolbox.NineML.NineMLModel-class.html]

xml version="1.0" encoding="ascii"?


PyDSTool.Toolbox.NineML.NineMLModel


| Home | Trees | Indices | Help | PyDSTool |
| --- | --- | --- | --- | --- |

Package PyDSTool ::
Package Toolbox ::
Module NineML ::
Class NineMLModel

[hide private]

[frames] | no frames]

# Class NineMLModel

source code

```
          object --+        
                   |        
ModelSpec'.ModelSpec --+    
                       |    
ModelSpec'.LeafComponent --+
                           |
                          NineMLModel
```

---


|  |  |
| --- | --- |
| Instance Methods | [hide private] |

**Inherited from `ModelSpec'.LeafComponent`**:
`add`,
`compileFuncSpec`,
`isEmpty`

**Inherited from `ModelSpec'.LeafComponent`** (private):
`_register`

**Inherited from `ModelSpec'.ModelSpec`**:
`__call__`,
`__contains__`,
`__copy__`,
`__deepcopy__`,
`__delitem__`,
`__eq__`,
`__getitem__`,
`__init__`,
`__ne__`,
`__repr__`,
`__str__`,
`addConnxnTarget`,
`delConnxnTarget`,
`difference`,
`flattenSpec`,
`info`,
`isComplete`,
`isDefined`,
`remove`,
`rename`,
`search`,
`validate`

**Inherited from `ModelSpec'.ModelSpec`** (private):
`_infostr`

**Inherited from `object`**:
`__delattr__`,
`__getattribute__`,
`__hash__`,
`__new__`,
`__reduce__`,
`__reduce_ex__`,
`__setattr__`


|  |  |
| --- | --- |
| Class Variables | [hide private] |

compatibleGens = `['Radau_ODEsystem', 'ADMC_ODEsystem', 'Vode_O...`

targetLangs = `['c', 'python', 'matlab']`

**Inherited from `ModelSpec'.LeafComponent`**:
`compatibleContainers`,
`compatibleSubcomponents`


|  |  |
| --- | --- |
| Properties | [hide private] |

**Inherited from `object`**:
`__class__`


|  |  |
| --- | --- |
| Class Variable Details | [hide private] |

### compatibleGens

Value:
:   ```
    ['Radau_ODEsystem',
     'ADMC_ODEsystem',
     'Vode_ODEsystem',
     'Euler_ODEsystem',
     'Dopri_ODEsystem',
     'ExplicitFnGen']
    ```

  


| Home | Trees | Indices | Help | PyDSTool |
| --- | --- | --- | --- | --- |

|  |  |
| --- | --- |
| Generated by Epydoc 3.0.1 on Fri May 4 15:24:08 2012 | http://epydoc.sourceforge.net |
